# Retention of first aid and basic life support skills in undergraduate medical students

**DOI:** 10.3402/meo.v19.24841

**Published:** 2014-11-06

**Authors:** Pim A. de Ruijter, Heleen A. Biersteker, Jan Biert, Harry van Goor, Edward C. Tan

**Affiliations:** 1 Institute for Scientific Education and Research, Radboud University Medical Center, Nijmegen The Netherlands; 2 Department of Surgery – Traumasurgery, Radboud University Medical Center, Nijmegen, The Netherlands

**Keywords:** basic life support, first aid, education, medical students, retention, skills

## Abstract

**Background:**

Undergraduate medical students follow a compulsory first aid (FA) and basic life support (BLS) course. Retention of BLS seems poor and only little information is provided on the retention of FA skills. This study aims at evaluating 1- and 2-year retention of FA and BLS training in undergraduate medical students.

**Methods:**

One hundred and twenty students were randomly selected from first year (*n*=349) medical students who successfully followed a compulsory FA and BLS course. From these 120 students, 94 (78%) and 69 (58%) participated in retention tests of FA and BLS skills after 1 and 2 years, respectively. The assessment consisted of two FA stations and one BLS station.

**Results:**

After 1 year, only 2% passed both FA and BLS stations and 68% failed both FA and BLS stations. After 2 years, 5% passed and 50% failed both FA and BLS stations. Despite the high failure rate at the stations, 90% adequately checked vital signs and started cardiopulmonary resuscitation appropriately.

**Conclusions:**

The long-term retention of FA and BLS skills after a compulsory course in the first year is poor. Adequate check of vital signs and commencing cardiopulmonary resuscitation retained longer.

## Introduction

The medical profession, health care providers, and the public expect junior medical doctors to be competent in first aid (FA) and basic life support (BLS) (1, 2). Training of FA and BLS in the medical curriculum is fundamental to achieve this competency (3).

One of the objectives at graduation, as stated in the Blueprint version 2009, is proficiency in FA and BLS. We demonstrated previously that a great diversity exists in form and content of FA and BLS training among the different medical schools in the Netherlands and that the training offered did not meet the criteria described in the directive for undergraduate medical education (1).

Several reports demonstrate medical students and junior doctors are not competent in providing BLS and suggest insufficient repetitive training and/or lack of skills retention as the cause of incompetency (3–8). Infrequently used skills deteriorate over time, and this deterioration increases over time. Psychomotor skills in resuscitation already decreased after 10 weeks in undergraduate nursing students (9). Other studies have shown that practical skills deteriorated mostly in the first month after training and stabilized after 1 year, but stayed well above pre-training levels (4, 9).

Also, different skills show different decay patterns. For example, procedural tasks show more decay than physical tasks, and declarative knowledge shows less decay than both physical and cognitive tasks. In order to maintain skills at an adequate level, repetitive training is required. Repetitive training needs to be tailored to the type of skill and to the interval with earlier skill acquisition. Ideally, retraining is initiated before skills drop below minimally acceptable levels and emphasis is on those skills that show greatest deterioration (10).

Whereas repetitive training has been mostly studied as a means of BLS skills acquisition, little information is provided on retention of elementary FA skills.

In parallel with the start of a medical bachelor (year 1–3) and master (year 4–6) curriculum in 2005 in Nijmegen, a new FA and BLS course has been developed for first year students at our medical school. This course has the objective to achieve competence at the end of the bachelor curriculum by repetitive training in FA and BLS skills. It aims to retrain before significant deterioration of skills has occurred and only to retrain those skills that deteriorate most.

This cohort study aimed at evaluating this approach of FA and BLS training by assessing the 1- and 2-year retention of FA and BLS skills in undergraduate medical students.

## Methods

### Study objectives

We conducted this cohort study to assess the retention of the skills trained in our course and to assess the methods used. In the long term, we aim to improve the course and to increase retention of skills.

### FA and BLS curriculum

**Fig. 1:**
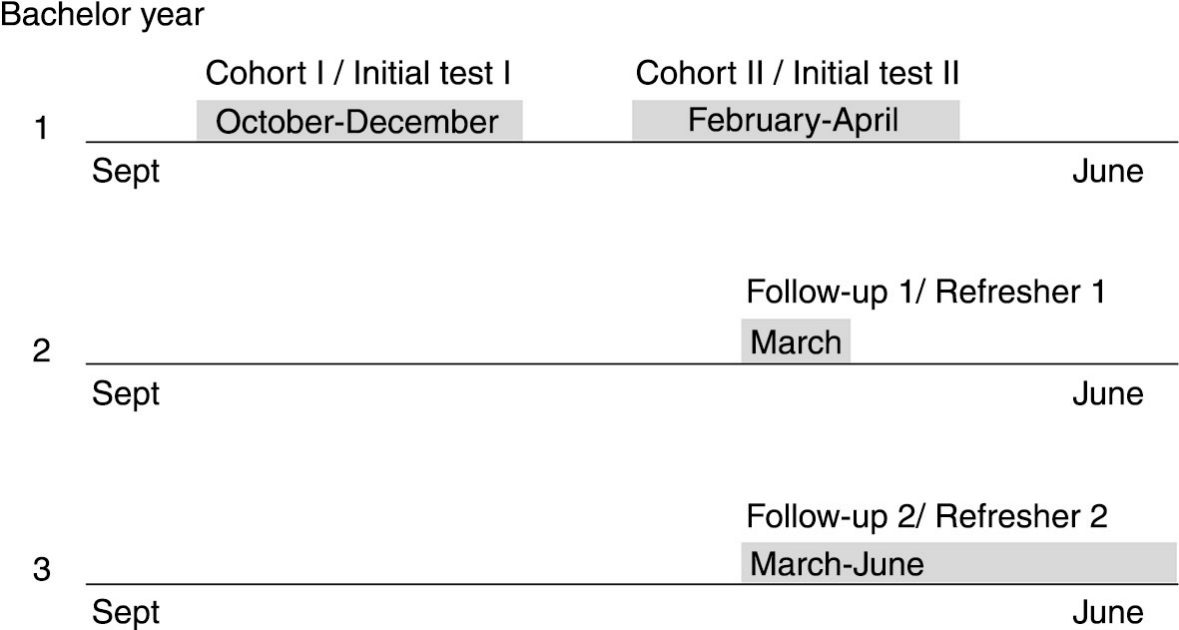
FA and BLS curriculum timeline.

The course is compulsory and consists of eight consecutive lessons of 2 hours each. Students are trained in small groups of 15 students and 2 student-instructors. The course emphasizes training on practical skills and procedural tasks. A systematic approach to check vital signs and treat life-threatening conditions using the ABCDE approach is taught (11, 12). The course is followed by a theoretical and practical examination meeting the requirements as stated in the blueprint. In the second bachelor year, a refresher course consisting of two sessions of 2 hours of FA and BLS is organized with a 5-month interval, and in the third year, pediatric basic life support (PBLS) is introduced during a 2-hour session in which the adult BLS skills are also refreshed (Fig. 1).

### Study group

Participants were first-year medical students attending the compulsory course in the academic year 2006–2007, divided into two cohorts (I and II) for organizational reasons. Cohort I received the initial training between October and December 2006 and cohort II received the initial training between February and April 2007. Format and content of the course were similar for both cohorts. Students were randomly assigned to follow the initial course in cohort I or cohort II by student administration of our medical school. One year after the initial course, a random sample of 120 was selected of the 349 students who had subscribed for the initial course in 2006–2007. Students were included if they had passed the exam of the initial course. Informed consent was obtained from students. According to the policy document of the institutional review board, ethical approval was not needed (http://www.ccmo.nl).

### Study design

We conducted a cohort study. Students were assessed by means of two FA and one BLS skills station, 1 year (Follow-up 1, FU1) and 2 years (Follow-up 2, FU2) after the initial course. The same test methods were used as in the initial course. Immediately after FU1, all students followed the second year compulsory refresher course in FA and BLS. Intervals between initial course, FU1, and FU2 are different between cohorts because of organizational reasons. The first FA station used a trained actor as a patient for assessment of vital signs using the ABCDE approach and to institute FA treatment. For this station, various scenarios were available such as trauma, anaphylaxis, myocardial infarction, and shock, to which the students were randomly allocated (Appendix). In the second FA skills station, the student had to demonstrate two practical skills such as FA bandage, recovery position, Heimlich maneuver, or treatment of an arterial bleeding (Appendix). The third station included a BLS scenario where the student had to demonstrate assessment of vital signs and subsequently perform 2 min of BLS on a resuscitation manikin, Ambuman C with Ambu CPR Printer (Ambu A/S Ballerup, Denmark) according to the guidelines of the European Resuscitation Council (ERC) 2005 (12) (Appendix).

Total duration of the assessment was 30 min, including time to change stations; each skills station had a maximum duration of 8 min. Students were randomly assigned to start with a FA or the BLS skills station. Theoretical knowledge was tested separately only in the initial course. In FU1 and FU2, only practical skills were assessed. Students were randomly assigned to one of the FA scenarios. The BLS scenario was equal for all students.

### Data collection

The following data were collected from the student registration department: sex, age, and previous academic courses within our university. Two trained student-instructors scored all skills stations at the initial course and at the follow-up tests, according to a standard checklist. For example, opening the airway will result in a performed or not performed check. The checklists and requirements used were developed based on the ABCDE approach and the ERC guidelines 2005 (12). Criteria for bandages were functionality and effectiveness; emergency maneuvers had to be adequately demonstrated according to the Orange Cross (13) (an important FA provider course for lay people in The Netherlands) and ERC guidelines (12). The scoring was as follows: all students started with a maximum amount of points (10 for the BLS station and 30 for the combined score of both FA stations). Points were subtracted each time a part of the assessment was not or incorrectly performed. Notably, this implies the possibility of a negative score (Appendix). Both FA skills stations were scored as one entity and are further referred to as FA. Students passed the FA station if their score was 10 or higher and the BLS station if their score was −1 or higher.

### Outcomes

Primary outcomes were passing or failing of the FA or BLS testing stations. There were four possible outcomes; passed all stations, passed FA station but failed BLS station, passed BLS station but failed FA station, and failed all stations. Success rates between FU1 and FU2 were compared.

Secondary outcomes were the separate scores at FA and BLS skills stations at FU1 and FU2. Scores of the initial course and FU1 were compared to observe the decline in scores; in addition, we compared the scores of FU1 and FU2 to investigate long-term retention. In students who failed skills stations at FU1 (BLS and/or FA), we checked if they performed an adequate assessment of vital signs, alerted emergency services, and to which degree they have met the requirements for performing adequate cardiopulmonary resuscitation. We compared cohort I and cohort II to analyze the effect of different time intervals between the initial course and FU1 and FU2.

### Statistical analysis

After the initial course, 345 students were available for follow-up. Power analysis showed that sampling 120 students would be sufficient. Our hypothesis was that 30% would pass the tests in follow-up I.

Student demographics and assessment scores were compared using an unpaired *t*-test in case of continuous variables, Pearson's chi-square tests for nominal variables and Mann–Whitney U test in case of ordinal variables or non-parametric distributed data. The Kruskal–Wallis test was used to compare the scores between the initial course and FU1 and FU2. All statistical analyses were performed using SPSS version 16.0 for windows. A *p*-value < 0.05 was considered statistically significant.

## Results

**Fig. 2:**
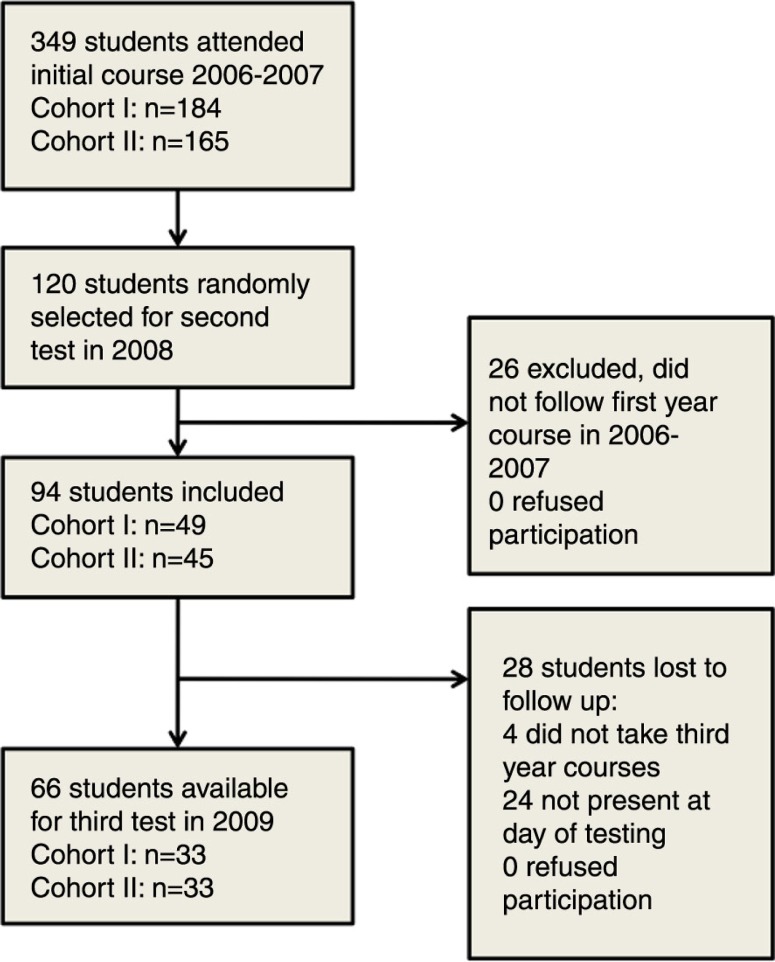
Study flow-chart.

Three hundred and forty-nine students passed the initial exam and were eligible for our study. Ninety-four of 120 randomly selected students (78%) were assessed at a median [range] of 11 [11–15] months (FU1) and 66 of the 94 (70%) at a median [range] of 26 [22–30] months (FU2). Reasons not to participate in the study are listed in Fig. 2.

There were no statistically significant differences in age and sex between all students within cohort I and cohort II and the FU1 and the FU2 groups. (Table 1)

**Table 1:** Student characteristics; gender, and age at inclusion

	First year (n = 349)	FU1 (n = 94)	FU2 (n = 66)	*p*
Gender, male	120 (35)	28 (30)	22 (33)	0.68^a^
Age at inclusion	18 (18–19)	18 (18–19)	18 (18–19)	0.29^b^

Data are displayed as n (%) or mean (interquartile range), statistics:

a chi square test,

b Kruskal Wallis.

FU1 = Follow-up 1, FU2 = Follow-up 2.

### Primary outcomes

At FU1, 2 of 94 students (2%) passed all skills stations, 11 (12%) passed the FA station, and 17 (18%) passed the BLS station. Sixty-four (68%) failed both skills stations. At FU2, 3 of 66 students (5%) passed both stations, 9 (14%) passed the FA station, and 21 (32%) passed the BLS station. Thirty-three (50%) failed both stations. The success rates of both stations at FU1 and FU2 were significantly lower (*p*<0.001) than the initial test. There were no significant differences in success rate between FU1 and FU2.

### Secondary outcomes

**Fig. 3:**
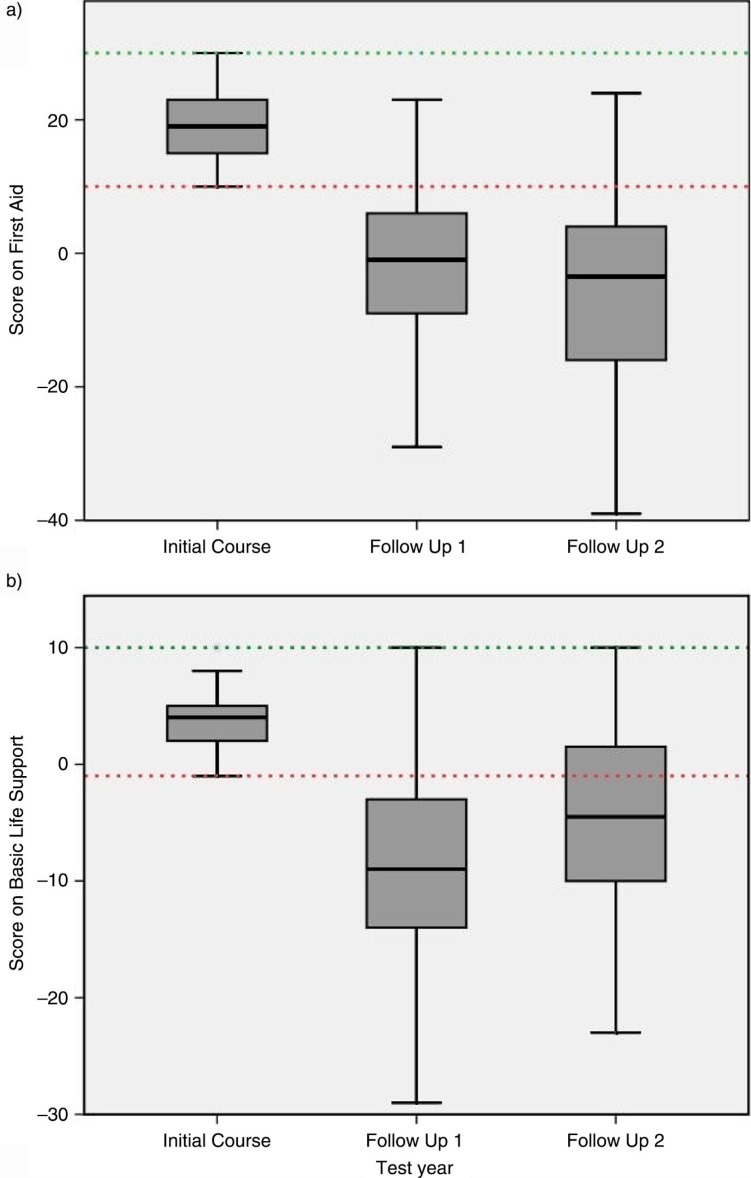
First aid and basic life support test results; group scores. A) Boxplot of scores on first aid. Dotted lines represent minimum score to pass of 10 and maximum achievable score of 30. B) Boxplot of scores on basic life support. Dotted lines represent minimum score to pass of –1 and maximum achievable score of 10.

Scores on the FA and BLS skills station are displayed in Fig. 3. Median score on the FA station at the initial assessment was 19 (interquartile range [IQR]: 15–26). FU1 and FU2 scores declined to a median of −1 (IQR: −9 to 6) and −4 (IQR: −16 to 4), respectively. Scores on the BLS station also declined over time; initial median score was 4 (IQR: 2–5), median FU1 score was −9 (IQR: −14 to −3), and median FU2 score was −5 (IQR: −10 to 2). The decline in scores on the FA and the BLS stations between the initial course, FU1, and FU2 were significant (*p*<0.001). Assessment of the score forms showed that more than 90% of students who failed the FA test in FU1 performed an adequate assessment of airway, breathing, and circulation.

At the BLS skills station in FU1, all students started cardiopulmonary resuscitation according to ERC guidelines when appropriate, 89% called the emergency services, and 75% performed adequate checks of vital signs. In 15%, a ‘faulty move’ was observed (e.g., incorrect hand placement), potentially doing further harm. Main reasons for failure at the BLS station were inadequate ventilation (volume below 400 mL) and inadequate compression depth (less than 40 mm or over 50 mm).

### Cohort analysis

Cohort I scored not significantly higher at the FA station (median 21, IQR: 16–24) compared to cohort II (median 18, IQR: 15–21, *p*=0.16) at the initial assessment; and at FU1 (cohort I: median 0, IQR: −7 to 7; cohort II: median −4, IQR: −11 to 5, *p*=0.049). Initial scores of BLS station were comparable between both cohorts (cohort I: median 5, IQR: 2–7; cohort II: median 2, IQR: 2–7, *p*=0.43). At FU1, students in cohort I scored significantly lower (median −10, IQR: −17 to −6) compared to cohort II (median −8, IQR: −13 to −1, *p*=0.04).

At FU2, there were no significant differences between cohorts for the FA and the BLS station (FA station: cohort I, median score −2; cohort II, median score −4, *p*=0.11; BLS station: cohort I, median score −3; cohort II, median score −5, *p*=0.25).

## Discussion

We studied the retention of FA and BLS skills in undergraduate medical students, undergoing our standard training protocol. FA and BLS skills deteriorate within 1 year. Almost all students failed the exam 1 year after the initial course but performance did not show any decay after 1 year. Skills to adequately check vital signs and start CPR when appropriate were preserved longer.

Our findings are consistent with literature, showing decreased retention of skills over time. Still, it should be taken into account that our study has a considerably longer follow-up, and interval time compared to other studies (8, 14, 15). As described in the introduction section, several studies show that practical skills in resuscitation decrease rapidly (4, 9). Although we used different outcome measures, and a different population, retention in our study seemed lower than previous studies show. Likely explanations are the lack of clinical practice in the first year, and the long time between the first and second course. Also, the strict criteria used for passing the exam compared to other studies might explain the poor retention percentages. In FU1, most students did adequately assess vital signs and called the emergency services, but failed to maintain sufficient chest compression depth and ventilation volumes; we believe this decay in practical skills is caused by the lack of opportunities for practice.

In comparison to nurses and doctors, undergraduate medical students have little opportunity to perform or observe FA or BLS skills in clinical practice (16, 17). In our curriculum, first exposure to clinical patients and emergency medicine is during the master curriculum, which results in low exposure to FA and BLS training opportunities. Jensen and colleagues, who examined the influence of clinical experience prior to an adult life support (ALS) course, also show the importance of clinical practice on skill retention. They demonstrated that having half a year of clinical experience had a significant impact on retention of both knowledge and skills at 6 months (18). Another study examined ALS skills retention in multi-disciplinary groups; the group that performed best at 6 months was found to have more direct care providers and more frequent ALS performers compared to the other groups (16). Repetitive simulation training, more frequently than practicing health care professionals, and early practical experience, for example, pre-hospital experience and training at a FA post, ambulance service, or emergency room, would benefit skills retention and should be adopted in current undergraduate medical curricula. However, we are aware of the amount of resources that may be required to implement such training.

Our study design did not allow for analysis of improvement of the different skills above pre-training level, because we did not assess pre-training level, and outcome at follow-up was either pass or fail of the skills station. Despite the poor retention found in this study, capability of delivering adequate FA was preserved in the majority of students. Knowledge more than psychomotor skills are needed for adequately checking vital signs and judgment to start resuscitation. Our tests are designed to assess the ability to reproduce the approach taught in our course. The high failure rates in FU1 and FU2 reflect that the majority of students were not able to reproduce all elements of the approach. However, assessment of the score forms used showed that elementary skills such as assessment of vital signs were preserved after 1 year and failure was due to insufficient practical skills of chest compression and ventilation. First and second year students probably retained their theoretical knowledge because they followed several modules on cardiovascular and respiratory physiology and disorders. We suggest that BLS and FA curricula should emphasize primarily practical skills and procedural tasks because these deteriorate most.

Factors other than time, such as age, ethnicity, and physical disabilities may have determined poor retention of skills (19). Age and sex were not significantly different between the two cohorts. We did not assess ethnicity and physical disabilities but would have identified disability compromising FA and BLS skills training.

Some studies attribute poor retention to inadequate coaching by the instructor during the practical lessons; for example, correcting skills inadequately during training (20, 21). This seems unlikely because all study participants passed the first year exam and did not receive training before FU1, although this is a matter of concern. Also, there were no significant differences in success rates between FU1 and FU2 despite the refresher course in between. This might be because the refresher course was immediately after the assessment and there was a large interval between the refresher course and FU2. Also, results in FU2 could have been worse without the refresher course.

This study has several limitations. Students had to be notified before the examination at 2-year follow-up. This inherited the potential risk of students preparing for the test. A separate study, however, showed that less than 10% prepared for the test and at subgroup analysis, exam results were comparable to those of unprepared colleagues. Scoring of the skill stations in FU2 was done by one observer, whereas in the initial test and FU1 two observers were used. We did not check for intra-class and inter-class variability. However, objective measures (e.g., prints from the manikin) were used for the BLS station to score the quality of compressions and ventilation; these scores were checked by two observers in the initial assessment, FU1 and FU2.

Based on our study results, we recommend shorter intervals for repetitive training and early exposure of undergraduate medical students to simulated and real life emergencies to improve retention of FA and BLS skills. We have also used the data from this study to improve the teaching capabilities of our student–instructor group.

## Conclusion

In undergraduate medical students, long-term retention of FA and BSL skills after following an extensive course is very poor. Skills to adequately check vital signs and to start cardiopulmonary resuscitation are preserved over time.

## Appendix

**FA station 1**

Case 1: Fractured skull base and epidural hematoma

Case description for student

A collision between your car and a cyclist just happened. The cyclist fell off his bike and is lying next to the car. You approach the cyclist to start first aid.

Case description for actor:

The victim is unconscious and responds only to physical stimulation. He complains of a moderate headache but responds adequately to questions. After the checks in Disability I, he loses consciousness again and does not respond to any stimuli. On inspection there is cerebrospinal fluid leakage from his nose.

Student Name: ________________________ Student Number: ____________________

Examinator: __________________________ Date: ______________________________

**Case 1:** Skull base fracture and epidural hematoma

	Correct				
				
Action	Y	N	Score (if No)	Remarks	Score
**Danger**	O	O	**−3**		
**Response:**					
Verbal approach	O	O	**−2**		
Gently shake shoulders	O	O	**−5**		
**Shout for help**	O	O	−**3**		
**Airway** check	O	O	−3		
**Breathing** check	O	O	−3		
**Circulation** check					
Radial artery (5–12 sec)	O	O	**−3**		
Checks capillary refill time or skin color and temperature	O	O	**−3**		
**Disability 1**					
Checks for signs of spinal cord injury: numbness, motor response, sensory response	O	O	Complete: **−8** Partial: **−4**		
Assess for loss of consciousness	O	O	**−3**		
Assess for amnesia	O	O	**−3**		
Inspection for CSF/blood in nose and ears	O	O	**−3**		
Checks orientation	O	O	**−1**		
**Calls emergency services**	O	O	**−10**		
**Exposure**					
AMPLE	O	O	**−0**		
Secondary survey	O	O	**−0**		
**Monitoring**					
Reassessment of airway patency	O	O	**−5**		
Reassessment breathing and circulation	O	O	**−4**		
**Faulty moves:**			**(if Y)**		
Unnecessary movement of victim	O	O	**−5**		
**Correct diagnosis:**					
Diagnosis correct?					
(Skull base fracture and epidural hematoma)			Only one correct: **−3** None correct: **−6**		

Total score: ______

**FA station 2**

Student Name: ________________________ Student Number: _____________________

Examinator: __________________________ Date: _____________________________

**Maneuver 1:** Recovery position

	Correct				
				
Action	Y	N	Score (if No)	Remarks	Score
Adequate support of head and neck while turning the victim	O	O	**−5**		
Adequate and stable position	O	O	**−5**		
Adequate sniffing position	O	O	**−2**		
**Faulty moves**					
Head drops on the floor while turning	O	O	**−5 (if Yes)**		

Score: ______ Student Name: ________________________ Student Number: _____________________

Examinator: __________________________ Date: _____________________________

**Bandage 2:** ankle pressure bandage

	Correct				
				
Action	Y	N	Score	Remarks	Score
Ankle in 90 degrees position	O	O	**−4**		
Correct use of materials	O	O	**−5**		
Used correct order of maneuvers	O	O	**−5**		
Applied bandage in proximal direction	O	O	**−3**		
Correct use of bandage	O	O	**−3**		
Ankle fixed stable	O	O	**−4**		
**Faulty moves**					
Bandage applied too tight	O	O	**−5**		
Less than 1 cm border of synthetic cotton wool to prevent pinching	O	O	**−2**		

Score: ______

**BLS station**

Student name: ________________________ Student number: _____________________

Examinator: __________________________ Date: _____________________________

**Table:** Adult Cardio Pulmonary Resuscitation Approach of the victim

	Correct				
				
Action	Y	N	Score if Not	Remarks	Score
**Danger**	O	O	**−3**		
**Response**					
Verbally assess responsiveness	O	O	−5		
Gently shake (one or both shoulders)	O	O	−5		
**Shout** for help	O	O	−5		
**Breathing**	O	O	−5		
Check during 5–10 sec	O	O	−3		
**Look, listen, feel while using chin lift**					
**Airway**					
Correct performance of chin lift	O	O	−5		
**Call emergency services (112)**	O	O	−5		
**Correct order**					
D-R-S-A-B-112-Co-Br-etc.	O	O	−3		
**Hand position**					
Both hands centered on the chest	O	O	**−3**		
**Posture**					
Above sternum with stretched arms	O	O	**−3**		
**Rescue breaths/ventilation**					
Observed chest rising	O	O	**−3**		

Score on this page: ______

Within every gray area, points can be subtracted only once.

**Table:**

Technical assessment		Score	Remarks	Score
**Time of compression cycle**				
<12 sec	O	**−7**		
<16 sec	O	**−3**		
Between 16–20 sec	O	**−0**		
>20 sec	O	**−3**		
>24 sec	O	**−7**		
*Assess third worst cycle for 30 chest compressions (18 sec)*				
**Time between compression cycles**				
<6 sec	O	**−0**		
Between 6–8 sec	O	**−3**		
>8 sec	O	**−10**		
*Assess third worst cycle, time from last compression of previous cycle to first compression of next cycle (6 sec)*				
**Compression: ventilation ratio**				
None or 1 cycle incorrect	O	**−0**		
> 1 cycle incorrect	O	**−3**		
*Compression: Ventilation (30:2)*				
**Compression depth**				
Less than 30 inadequate	O	**−0**		
More than 30 inadequate *(adequate 40–50 mm)*	O	**−5**		
**Ventilation volume**				
Less than 2 inadequate	O	**−0**		
More than 2 inadequate	O	**−5**		
Gastric insufflation *(adequate 0.4*–*0.8 L)*	O	**−5**		
**Recoil of chest during compressions**				
Complete recoil	O	**−0**		
< 20 compressions incomplete recoil	O	**−0**		
> 20 compressions incomplete recoil	O	**−3**		

Score: ______

**Total score on both pages: ______**

## References

[1] Draaisma JMT, Roest G, van Kesteren RG, Vulto A (2006). Inventarisatie van het onderwijs in de spoedeisende geneeskunde in de opleiding tot basisarts in Nederland. Tijdschr voor Med Onderwijs.

[2] Laan RFJM, Leunissen RRM, van Herwaarden CLA (2009). The 2009 framework for undergraduate medical education in The Netherlands. http://www.vsnu.nl/Media-item/Raamplan-Artsopleiding-2009.htm.

[3] Das M, Elzubeir M (2001). First aid and basic life support skills training early in the medical curriculum: curriculum issues, outcomes, and confidence of students. Teach Learn Med.

[4] Wilson E, Brooks B, Tweed WA (1983). CPR skills retention of lay basic rescuers. Ann Emerg Med.

[5] Ahmed HU, Kellett C, Ashworth M, Nazir S (2004). First aid and cardiopulmonary resuscitation training for medical students. Med Educ.

[6] Sternbach GL, Kiskaddon RT, Fossel M, Eliastam M (1984). The retention of cardiopulmonary resuscitation skills. J Emerg Med.

[7] Tan EC, Severien I, Metz JC, Berden HJ, Biert J (2006). First aid and basic life support of junior doctors: a prospective study in Nijmegen, the Netherlands. Med Teach.

[8] Woollard M, Whitfeild R, Smith A, Colquhoun M, Newcombe RG, Vetteer N (2004). Skill acquisition and retention in automated external defibrillator (AED) use and CPR by lay responders: a prospective study. Resuscitation.

[9] Madden C (2006). Undergraduate nursing students’ acquisition and retention of CPR knowledge and skills. Nurse Educ Today.

[10] Wisher RA, Sabol MA, Ellis J Staying sharp: retention of military knowledge and skills.

[11] (2008). Advanced Trauma Life Support for doctors.

[12] Handley AJ, Koster R, Monsieurs K, Perkins GD, Davies S, Bossaert L (2005). European Resuscitation Council guidelines for resuscitation. Section 2. Adult basic life support and use of automated external defibrillators. Resuscitation.

[13] Spruyt VM, De DBV (2006). Het Oranje Kruis. Oranje Kruis Boekje, Officiele handleiding tot het verlenen van eerste hulp bij ongelukken [The Orange Cross. Orange Cross Booklet, The Official First Aid Guide].

[14] Fossel M, Kiskaddon RT, Sternbach GL (1983). Retention of cardiopulmonary resuscitation skills by medical students. J Med Educ.

[15] Wynne G (1986). ABC of resuscitation. Training and retention of skills. Br Med J (Clin Res Ed).

[16] Smith KK, Gilcreast D, Pierce K (2008). Evaluation of staff's retention of ACLS and BLS skills. Resuscitation.

[17] Hammond F, Saba M, Simes T, Cross R (2000). Advanced life support: retention of registered nurses’ knowledge 18 months after initial training. Aust Crit Care.

[18] Jensen ML, Lippert F, Hesselfeldt R, Rasmussen MB, Mogensen SS, Jensen MK (2009). The significance of clinical experience on learning outcome from resuscitation training-a randomised controlled study. Resuscitation.

[19] Riegel B, Birnbaum A, Aufderheide TP, Thode HC, Henry MC, Van Ottingham L (2005). Predictors of cardiopulmonary resuscitation and automated external defibrillator skill retention. Am Heart J.

[20] Kaye W, Rallis SF, Mancini ME, Linhares KC, Angell ML, Donovan DS (1991). The problem of poor retention of cardiopulmonary resuscitation skills may lie with the instructor, not the learner or the curriculum. Resuscitation.

[21] Parnell MM, Larsen PD (2007). Poor quality teaching in lay person CPR courses. Resuscitation.

